# Determinants of eating behaviour in university students: a qualitative study using focus group discussions

**DOI:** 10.1186/1471-2458-14-53

**Published:** 2014-01-18

**Authors:** Tom Deliens, Peter Clarys, Ilse De Bourdeaudhuij, Benedicte Deforche

**Affiliations:** 1 Department of Human Biometry and Biomechanics, Vrije Universiteit Brussel, Pleinlaan 2, 1050 Brussels, Belgium; 2 Department of Movement and Sports Sciences, Ghent University, Ghent, Belgium

**Keywords:** Determinants, Eating behaviour, University students, Focus groups

## Abstract

**Background:**

College or university is a critical period regarding unhealthy changes in eating behaviours in students. Therefore, the purpose of this study was to explore which factors influence Belgian (European) university students’ eating behaviour, using a qualitative research design. Furthermore, we aimed to collect ideas and recommendations in order to facilitate the development of effective and tailored intervention programs aiming to improve healthy eating behaviours in university students.

**Methods:**

Using a semi-structured question guide, five focus group discussions have been conducted consisting of 14 male and 21 female university students from a variety of study disciplines, with a mean age of 20.6 ± 1.7 yrs. Using Nvivo9, an inductive thematic approach was used for data analysis.

**Results:**

After the transition from secondary school to university, when independency increases, students are continuously challenged to make healthful food choices. Students reported to be influenced by individual factors (e.g. taste preferences, self-discipline, time and convenience), their social networks (e.g. (lack of) parental control, friends and peers), physical environment (e.g. availability and accessibility, appeal and prices of food products), and macro environment (e.g. media and advertising). Furthermore, the relationships between determinants and university students’ eating behaviour seemed to be moderated by university characteristics, such as residency, student societies, university lifestyle and exams. Recommendations for university administrators and researchers include providing information and advice to enhance healthy food choices and preparation (e.g. via social media), enhancing self-discipline and self-control, developing time management skills, enhancing social support, and modifying the subjective as well as the objective campus food environment by e.g. making healthy foods price-beneficial and by providing vending machines with more healthy products.

**Conclusions:**

This is the first European study examining perceived determinants of eating behaviour in university students and collecting ideas and recommendations for healthy eating interventions in a university specific setting. University characteristics (residency, exams, etc.) influence the relationships between individual as well as social environmental determinants and university students’ eating behaviour, and should therefore be taken into account when designing effective and tailored multilevel intervention programs aiming to improve healthy eating behaviours in university students.

## Background

Prevention of overweight and obesity, and its related diseases [1], has become a worldwide challenge [2]. According to US literature, university is a critical period for weight gain [3-5]. During the transition from secondary school to university, students need to adapt to a new environment [6,7]. When students fail to adapt adequately this could have negative consequences towards their health behaviours and subsequent weight status [7]. Eating behaviour (next to physical activity and sedentary behaviour) is an important factor influencing students’ weight. According to studies conducted in US universities, students were not eating the recommended amount of fruit and vegetables, and were consuming increasing amounts of high-fat foods [8-10]. Furthermore, Butler et al. [9] reported significant decreases in the amount of bread and vegetables consumed during the first year of university and significant increases in percentage fat intake and alcohol consumption in US students. Unhealthy eating and excessive alcohol consumption may contribute significantly to energy intake and may therefore facilitate student weight gain [11]. The same pattern of weight gain in university students is emerging in Europe [12]. However, European literature on dietary intake in university students is scarce. In a Greek study [13] university students showed significantly higher intake of total and saturated fat and lower intake of poly and monounsaturated fat, folate, vitamin E and fibre, compared to the American Heart Association guidelines. Crombie et al. [3] warned that these health behaviours may not only occur during the years at university but may remain throughout adulthood as well. Therefore, prevention programs countering unhealthy eating habits in university students are needed, in order to prevent an increasing prevalence of overweight and obesity in later life.

To develop effective obesity prevention strategies it is important to get insight into factors influencing eating behaviours in university students. Early theories explaining health behaviour mostly focused on the individual within its social context [14,15]. According to Ajzen’s Theory of Planned Behaviour [14], behaviour can be explained through intention. These intentions are being determined by attitudes toward the behaviour, social or subjective norms and perceived behavioural control [14]. The Social Cognitive Theory of Bandura [15] on the other hand, focuses on the interaction between personal (self-efficacy), behavioural (expected result) and social (modelling and social support) factors to explain health behaviours such as eating behaviour. Next to these psychosocial determinants, many researchers [16-18] are convinced of the uttermost importance of the environmental influence on eating behaviours. According to Brug et al. [16] the environment has obviously changed during the last decades, whereas opportunities to eat energy-dense foods are omnipresent. Egger et al. [17] suggested that the increasing obesogenic environment is the driving force for the increasing prevalence of obesity rather than any ‘pathology’ in metabolic defects or genetic mutations within individuals. Individuals interact in a variety of micro-environments or settings (e.g. schools, workplaces, homes, (fast food) restaurants) which, in turn, are influenced by the macro-environments or sectors (e.g. food industry, government, society’s attitudes and beliefs) [18]. Ecological models consider the connections and the continuous interactions between people (intrapersonal) and their (sociocultural, policy and physical) environments [19-21]. Based upon the latter two theories Story et al. [19] proposed a framework including individual (intrapersonal), social (interpersonal) environmental, physical environmental and macro levels, to understand factors influencing eating behaviours.

Only few qualitative studies, using focus group discussions, have examined determinants of eating behaviour in university students. Lack of discipline and time, self-control, social support, product prices (costs) and limited budgets, and the availability of and access to (healthy) food options were reported as important influencing factors of students’ eating behaviours [22-25]. All of these studies were conducted in the US and either not included students of all study disciplines [22,23] or did not specify students’ study backgrounds [24,25]. In addition, all studies included predominantly freshman students. However, including students of all study disciplines as well as students with more university experience (i.e. older students) could contribute to a wider range of experiences and opinions. Furthermore, no previous studies have included questions asking for recommendations towards intervention strategies aiming to improve healthy eating behaviours in university students. To the best of our knowledge, no European (qualitative or quantitative) studies on determinants of eating behaviour in university students have been conducted so far. Many differences in lifestyle, environment and culture (e.g. fast food culture) can be observed between North-American and European students. Continental differences as such might not only influence students’ eating behaviour but also the enablers and barriers to engage in healthy eating practices. Hence, there is a need for European studies investigating determinants of eating behaviour in university students. Therefore, the purpose of this study was to explore which factors influence Belgian (European) university students’ eating (incl. drinking) behaviour, using a qualitative research design. Furthermore, we aimed to collect ideas and recommendations in order to facilitate the development of effective and tailored intervention programs aiming to improve healthy eating (incl. drinking) behaviours in university students.

## Methods

### Participants

In this qualitative study focus group discussions were used for data collection. To ensure sufficient diversity of opinion, students from the second till fifth year of university from different study disciplines were recruited using snowball sampling, a purposive nonprobability approach that is often used in qualitative research and in which the researcher recruits a few volunteers who, on their turn, recruit other volunteers. No first year students were included because of their ‘limited’ experience as a university student. The aim was to recruit between six and ten participants per focus group [26]. An over-recruitment of one or two participants was pursued in case there were ‘no-shows’.

### Procedure

Focus groups were held until saturation of new information was reached, as in qualitative research sample size can never be pre-determined [26]. To be sure we did not miss any ‘new’ information, one additional focus group session was held after theoretical saturation was estimated. All focus groups were organised at the Faculty of Physical Education and Physiotherapy of the Vrije Universiteit Brussel (Brussels, Belgium) at a time and date convenient for the students and researchers. Before each focus group all participants were asked to complete a short questionnaire, including demographics, height, weight and perceived health (see Table 1). Furthermore, explanation about the aim of the study was given and an informed consent (in which participants’ anonymity and confidentiality were assured) was signed by each participant. Each focus group lasted between 90 and 120 minutes (including questions about physical activity and sedentary behaviour which were not included in this paper) and was facilitated by a moderator and an assistant moderator (observer), who took notes during the discussions and made sure the moderator did not overlook any participants trying to add comments. All focus group discussions were audiotaped with permission of the participants. Drinks and snacks were provided during the focus group discussions. Afterwards, all students received an incentive (a lunch voucher). The study was approved by the Medical Ethical Commission of the university hospital.

**Table 1:** Characteristics of focus group participants (Mean ± SD, %, n = 35)

Gender (% of females)	60.0
Age (yrs)	20.6 ± 1.7
Body Mass Index (BMI) (kg/m^2^)	22.8 ± 3.9
Underweight (%)	8.8
Normal weight (%)	61.8
Overweight (%)	29.4
Study career (yrs)	3.0 ± 1.0
Study discipline	
Human sciences (%)	62.9
Exact and applied sciences (%)	17.1
Biomedical sciences (%)	20.0
Residency (% living in student residence)	45.7
Smoking (% smokers)	11.4
Self-reported health (% reporting poor to very poor health status)	14.3
Perceived physical activity level (% reporting little to no physical activity)	48.6
Perceived eating pattern quality (% reporting poor to very poor eating pattern)	17.1

### Question guide

According to recommended focus group methodology [27], a semi-structured question guide (see Table 2) was developed by the research team, aiming to identify factors influencing university students’ health and weight related behaviours (including eating (and drinking) behaviour, physical activity and sedentary behaviour). As mentioned before, this paper will only focus on determinants of students’ eating behaviour. After intensive collaboration with experts with ample focus group experience, the questions were carefully developed using appropriate literature [27]. When development was completed, the question guide was tested within and revised by the research team as well as pilot-tested in a group of ten university students. Because no major changes had to be made, ‘pilot’ discussion results were included in later analysis [26]. The question guide consisted of opening and introductory questions which allowed participants to get acquainted and feel connected, and to start the discussion of the topic. Transition and key questions were used to, respectively, guide the group towards the main part of the discussion and to focus on the purpose of this study, i.e. identifying factors influencing students’ eating behaviour. For obvious reasons, the greatest share of the group discussions focused on the key questions. Finally, students were asked to share ideas concerning health promotion as well as intervention strategies to counter unhealthy eating behaviours in university students. During the focus group discussions the moderator followed the question guide but asked side questions to obtain more in-depth information about the topics, and showed enough flexibility to allow open discussions between students.

**Table 2:** Focus group question guide

**Question type**	**Question**
Opening	1. Where are you from and what’s your name?
Introduction	2. Describe a healthy person.
Transition	3. Thinking of ‘health in university students’, what comes to your mind?
	4. Think back of the last year(s) being a university student. Did your body weight and/or body composition change since you entered university?
Key	5. Did your health related habits change since you entered university?
	6. Which factors have caused these changes (or which factors influence current health behaviours)? What barriers and enablers of healthy behaviour can you identify?
	7. Which of the previous mentioned factors have had the greatest influence?
Ending	8. Do you have any remarks, suggestions, additions?
	9. Soon, we will try to help students make healthier choices. Can you give us some advice on how to promote healthy eating behaviours in students?

### Data analysis

SPSS Statistics 20 was used to analyse data obtained from the questionnaire and to calculate descriptive statistics of the focus group sample. Data obtained from the audio tapes where transcribed verbatim in Microsoft Word using Express Scribe and Windows Media Player. All quotes were encoded using the qualitative software program Nvivo9. Using an inductive thematic approach, data (quotes) were examined for recurrent instances of some kind, which were then systematically identified across the data set, and grouped together by means of a coding system (= content analysis) [28]. Similar codes were grouped together into more general concepts (subcategories) and further categorised into main categories. To ensure reliability of data interpretations, analyses were carried out independently by two researchers. Doubts or disagreements were discussed with two other researchers until consensus was reached.

## Results

In this study, the estimated point of saturation was observed after the fourth focus group session. One additional focus group discussion was conducted to be sure true saturation was established. In total, five focus group discussions have been conducted, consisting of five to ten participants per group. The sample (n = 35) consisted of 14 male and 21 female students with a mean age of 20.6 ± 1.7 yrs (range = 18-26 yrs) and a mean study career of 3.0 ± 1.0 yrs. The majority of students (62.9%) were enrolled in human sciences, whereas respectively 17.1% and 20.0% were enrolled in exact and applied, and biomedical sciences. Additional sample characteristics are described in Table 1.

**Figure 1:**
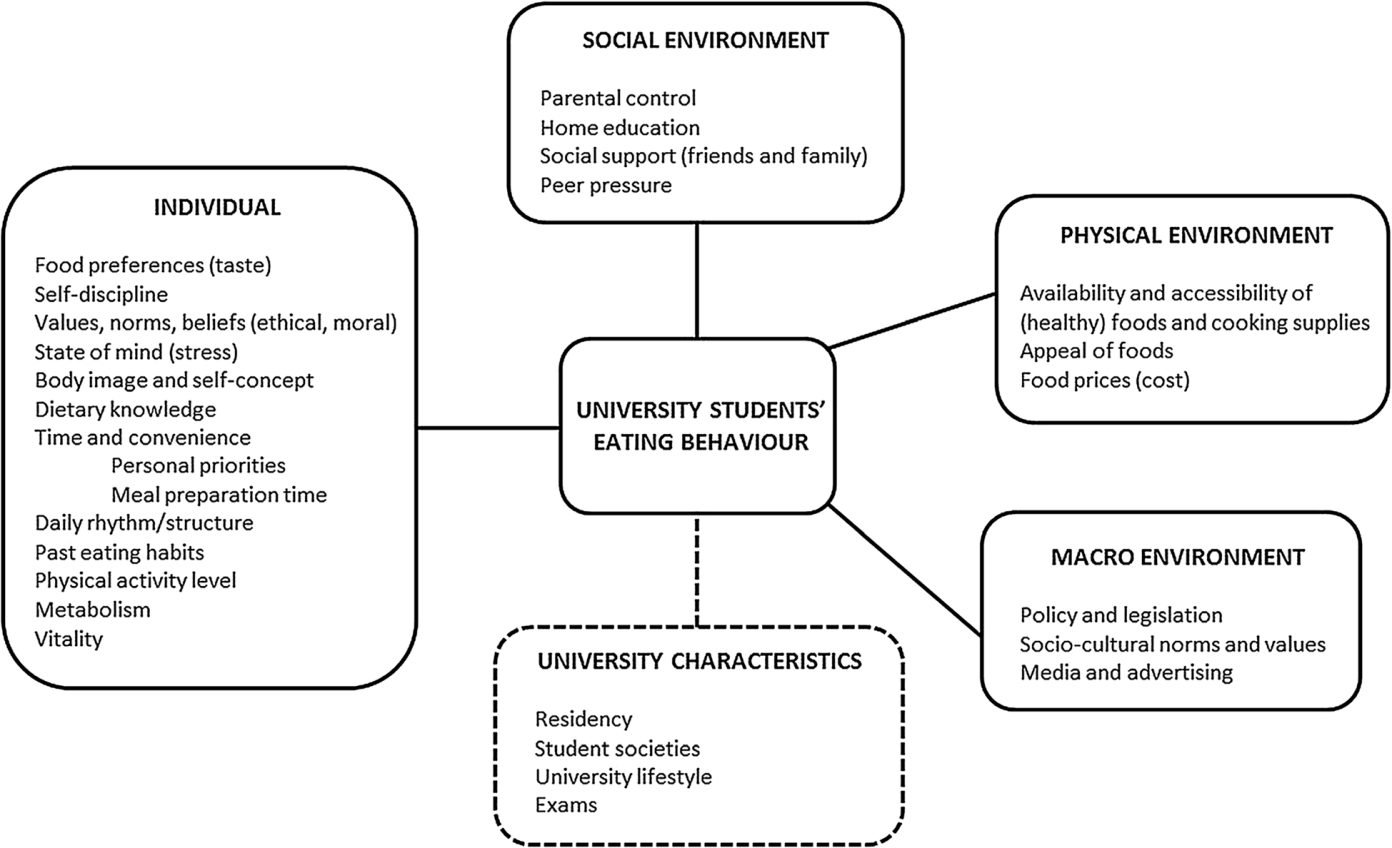
Factors influencing eating behaviours of university students.

According to the ecological principles a framework of factors influencing eating (incl. drinking) behaviours in university students was developed based on content analysis of the focus group discussions (Figure 1). The framework consists of four major levels, i.e. individual (intrapersonal), social environment (interpersonal), physical environment (community settings), macro environment, and an additional level of university characteristics. The most appropriate quotes were chosen to illustrate each (sub)category.

### Individual (intrapersonal)

#### *Food preferences (taste)*

Students reported that ‘taste’ is an important factor influencing their food choices. Taste can make students eat unhealthy, however it can help them make healthy choices as well: *“I choose to eat fruit because I like fruit”.*

#### *Self-discipline*

Students believed that self-discipline is related to self-dependency (autonomy) and may have an influence on their eating behaviour: *“I do think that self-discipline is an important factor (regarding eating behaviour) when you become self-dependent” (…) “You have to take care of yourself; some can and others can’t”.*

#### *Values, norms, beliefs (ethical, moral)*

According to the participants, norms and values as well as personal beliefs can influence students’ eating behaviours. One student explained that moral conviction had driven him to become a vegetarian: *“From the moment I became a vegetarian, it was so obvious for me that I didn’t have the need to eat meat anymore (…) Yes, this was a moral conviction and I didn’t have to have discipline for it because it seemed obvious to me”.* Students also explained that they sometimes changed their eating behaviours due to a feeling of guilt when eating unhealthy foods such as pizza.

#### *State of mind (stress)*

Students experienced the transition from secondary school to university as a stressful period. Participants also revealed that exam periods (when academic achievement pressure is highest) provide a lot of stress. Participants strongly believed that eating choices during stressful periods can be influenced in both directions. Some tend to eat healthier: *“I consume more during exam periods, but I tend to eat more fruits and vegetables”.* Others’ eating patterns tend to worsen when experiencing such ‘high’ stress levels: *“During exam periods I can eat ‘everything’; I’m always hungry”.* Not only academic stress, but also social stress can alter students’ eating behaviours: *“Yes, when you don’t feel well, e.g. heart broken, then the cliché of eating ice cream in front of the television becomes reality”.*

#### *Body image and self-concept*

Students spoke about their own body image and how it can have an effect on their eating behaviours. “*When you don’t find yourself attractive, consequently you think others will think the same. That’s a vicious circle and it keeps getting worse and worse. And this can influence someone’s eating behaviour.”* Students felt that body image is related to the socio-cultural ideal image and is, in turn, related to media advertisement strategies.

#### *Dietary knowledge*

Participants believed that a certain dietary knowledge is needed to be able to make changes in one’s eating pattern. To a certain extent students seemed to be aware of what is good for their health: *“Actually, I don’t like vegetables, but I know that I need it and that’s why I eat vegetables”.* However, they also stated that knowledge is just a first step and will not automatically lead to healthier food choices. *“When I would follow a health class tomorrow, it doesn’t necessarily mean I would suddenly change my eating behaviour.”*

#### *Time and convenience*

Time seems to be a very precious issue when talking about student eating practices. Students indicated they would rather spend time on other activities than cooking, especially when they have to cook only for themselves. Participants acknowledged that ‘time’ is a relative term and it is often related to personal priorities: *“Because here on campus (when living in a student residence) I always have something else to do instead of cooking, so I don’t have time to make dinner”*. Students explained that meal preparation time is of great importance: *“The faster my meal is ready, the better, so I can install myself in front of the television”.* According to the students easiness and convenience, which is related to time, is an important factor as well: *“I want it to be easy, so I don’t have to be cooking for one hour for myself, …, so I grab something that can be warmed up quickly”.* Time is mentioned to be especially important during exam periods: *“After exam periods, you have more time to cook. When you are studying (during exam periods), you want to spend as little time as possible on cooking”.*

#### *Daily rhythm/structure*

Students indicated that many students live a rather unstructured life (incl. sleeping habits), especially when living in student residences. Hence, their eating practices can suffer from this. *“When you stay awake longer, the urge to grab something sweet (cookies, candy, …) is bigger, whilst when having a good sleeping pattern, the urge may be smaller.”* On the other hand, when living with their parents, students felt they were subject to a certain ‘structure’. *“When I lived at home, everything was nicely structured and I didn’t even have to think about it, because the foundations had already been set by my parents.”*

#### *Past eating habits*

Participants felt they had regular eating habits. According to the students these eating practices are a result of eating habits created during childhood and adolescence: *“My healthy habits didn’t change by living away from home (in a student residence), because already all my life I drink 1.5 litres of water per day and I still do. I almost never drink Coca Cola.”*

#### *Physical activity level*

Students stated that a higher caloric intake is needed when exercising: *“I didn’t feel safe anymore when playing rugby, so I started eating more; more carbohydrates etc., so I pay attention to what I eat”*. Also, it was mentioned that some students tend to think they can eat anything they want after exercising: *“Some think that after they have exercised, they can eat a hamburger”.*

#### *Metabolism*

Students suggested that metabolism can differ between one another. Some students tend to ‘burn’ calories more easily than others. *“(One of the students towards a student colleague:) Your metabolism is abnormal, you eat and you drink what you want and you don’t gain weight.”*

#### *Vitality*

It was mentioned that when being tired, students tend to eat more energy-dense foods: *“When you are more tired the urge for sugars is bigger because you want to elevate your energy level”.* However, lack of vitality could also trigger some kind of health awareness: *“I noticed that in my first year at university I didn’t eat vegetables sufficiently and I felt tired and didn’t have enough energy. Therefore, I started eating vegetables and fruit. Consequently, I had lots more energy …”*

### Social environment (interpersonal)

#### *Parental control*

Students felt that parental control had a crucial role in their eating behaviours. When parental control is lacking this can have great influences on individual food choices. *“After the transition from secondary school to university, parental control decreased, so consequently ‘freedom’ increased, which means you become more self-dependent and that has influenced my eating behaviour. For example, in secondary school you had home prepared sandwiches for lunch, while at university you can eat cafeteria sandwiches and dessert etc.”* Moreover, alcohol consumption was described to be more common when parental control is lacking: *“In student residences it is easier to play drinking games, because your parents cannot see what you’re doing”.*

#### *Home education*

Students indicated that eating habits may depend on their home education. When one is raised in a more healthy environment it is more likely one consumes e.g. sufficient fruits and vegetables. *“My mother made me eat my vegetables, even though I didn’t like to.”*

#### *Social support (friends and family)*

Students revealed that support from family and friends can influence their eating behaviour: *“During exam periods I am happy that mom prepares my meal, because if I had to make it all by myself, I would make pizza more often”.* Living together with peers can also influence eating behaviours: *“I live together with my girlfriend, but when I would live all by myself, I would pay less attention to what I eat”.* Students emphasized the importance of a great social supporting network: *“When you have no friends, you can’t deal with ‘stressy’ events properly which can have direct consequences on your personal health”.*

#### *Peer pressure*

Group or peer pressure was explained to be an influencing factor of individual food choices. *“You can make your own sandwiches, but then you might be the only one in the group, so the next day chances are big you also buy a sandwich on campus.”*

### Physical environment (community settings)

#### *Availability and accessibility of (healthy) foods and cooking supplies*

When students have easy access to (on-campus) eating facilities, they seem to get tempted more easily. For example, the student restaurant and its meal offers seem to influence students regarding their individual food choices. *“In the student restaurant you can choose every day between French fries, (mashed) potatoes or rice. I think lots of students tend to take French fries every time.”* On the other hand students expressed that the university restaurant offers a lot of meal choices and it depends on the individual whether healthy or less healthy choices are being made. *“I think we cannot complain of what is offered in the student restaurant. There is enough variety.”* Participants also mentioned the presence of numerous candy machines on campus which might be of influence as well. On the other hand they mentioned that when healthy foods are available in their nearest environment, and certainly when it’s for free, they tend to eat more healthy alternatives. *“During exam periods free fruit was available in the university’s study hall and subsequently you could notice an increase of fruit consumption.”* Moreover, living in a student residence, where the availability of cooking supplies is often limited, can influence meal choices as well: *“Not all student residences have a fully equipped kitchen”* (…) *“Since I don’t have a fryer (in the student residence) I can’t prepare anything fried, so I mostly eat more healthy foods”.* Students also believed that a lack of cooking supplies could contribute to more unhealthy food choices.

#### *Appeal of foods*

Students believed that the appeal of food items makes it sometimes hard for them to make healthy choices. *“Indeed, the (student) restaurant is a ‘dangerous’ place, you walk into the canteen and you see others (friends) eating lasagne and subsequently you leave your sandwiches in your bag and go get some lasagne too. In contrast to secondary school, you get tempted more quickly now.”*

#### *Food prices (cost)*

Food product prices and individual budget influence students’ food choices. On the one hand, when eating outdoors, they might spend more money: *“If I’d buy a sandwich every day it would become too expensive. I live currently by myself and it (money/price) becomes more important, so I have to pay attention and eat my home prepared and healthier sandwiches”.* On the other hand, students also believed that unhealthier foods in e.g. fast food restaurants are less expensive than preparing a healthy meal at home. *“It will be more expensive when you eat healthy; for example a lasagne is cheaper than buying leek, onions and carrots.”* Students referred to the American eating culture: *“I think that unhealthy foods are cheaper than healthy foods. Just look at the ‘one dollar menus’ in the US; this is a big problem (regarding public health)”.* In contrast, others believed that this is not always true. “*Some vegetables and fruits are cheaper than certain cookies. So, that’s also one of the reasons why I sometimes buy apples (instead of cookies); because it’s cheaper.”* Participants also mentioned that when living in a student residence, one becomes more self-dependent which also implies that price and budget become more and more important. *“When you live in a student residence, you have to buy your own food, so you automatically start to pay attention to product prices.”*

### Macro environment

#### *Policy and legislation*

Students recognised that they are restricted by policy and legislation which influence their drinking choices. E.g. alcohol consumption can be influenced by governmental regulations: *“When you go out by car, you are not allowed to drink and drive, so you will automatically drink less, … much less”.*

#### *Socio-cultural norms and values*

Students mentioned that certain eating behaviours can be region as well as society specific: *“But this (eating behaviour) is specific to our society; nowadays, when you look at the US, for them it is normal to go eat fast food every day, whilst here in Europe it isn’t”.* These socio-cultural norms do not only differ geographically, but can change over time as well: *“Our culture has evolved as such that alcohol has become a socially accepted drug”.*

#### *Media and advertising*

Participants felt influenced by media and advertising: *“When I see food on television, I am more likely to go get something from the cupboard; on the one hand because I feel like it, but also because I see it on television”.*

### University characteristics

#### *Residency*

Participants felt that students living in a student residence and being surrounded by other student peers are often subject to lots of stimuli influencing their eating behaviour. *“You see a lot of students who just arrived at university and stay in a student residence, eating lasagne, or pizza, …” (…) “I noticed that students living in student residences eat much more unhealthy foods, go out more and drink more …”* One students’ personal experience confirmed the latter: *“I have lived in a student residence for four years and I gained 10 kg of weight because of going out too much and eating unhealthy”.* However, other participants reported no changes in eating behaviour: *“I already live two years in a student residence and I don’t have the impression that my habits have changed, I still eat healthy”.* The student environment can have a positive influence on eating behaviour as well. Students expressed that when living in a student residence and cooking together (with peers) they take their time to prepare a meal which enlarges the chances of making a ‘healthy’ meal.

#### *Student societies*

Student societies influence students’ drinking habits: *“When you go out with other student society members, you are almost obliged to drink (alcohol)”.*

#### *University lifestyle*

Students explained that the excitement and novelty when arriving at university can cause students to go out more and ‘taste’ the university life. They also believed first year students arriving at university can be very influenceable: *“Many people I had never seen drinking before started drinking at university”.* Furthermore, one of the students explained how life at the university influenced her eating and drinking behaviour: *“When I arrived at university, I was a top athlete (swimming) and I had a very healthy lifestyle back then. When I quit (swimming) I practically lived on campus, so every night I went to parties and drank and ate a lot and my body experienced these changes. After my sporting career I ‘discovered the world’ (in terms of drinking, eating, friends, …) but now it has stabilised”.*

#### *Exams*

Participants reported that eating behaviours during the academic year can differ (in a positive and a negative way) from those during exam periods: *“During exam periods I gain weight, because I tend to quickly grab something during a break”.* In contrast, one of the students replied as follows: “*I eat healthier (during exam periods) to maintain my personal health, and I sleep more as well”.*

### Suggestions for interventions

#### *Individual level*

Participants believed that direct (one-on-one) communication should be used. *“You have to confront students individually (to sensitize), because ‘general’ promotion is not as effective (…) Giving students personal feedback on their health status will be more effective.”* Nevertheless, one student suggested to use posters: *“I think using posters displaying e.g. the ‘healthy eating pyramid’ will not stay unnoticed”*. Furthermore it was mentioned that *“all students should be given like one hour of information (about healthy eating) by means of a health class”*. However, one of the students mentioned that *“knowledge helps you to make decisions, but it doesn’t force you”*. Students also thought it was important to give advice via internet and social media: *“… like Facebook, you can check your messages whenever you want and you are free to choose whether you read it or not”*. Furthermore, participants believed that promotion strategies should focus on convenience: *“Promotion strategies should be ‘easy going’ and convenient, don’t make it look like students have to do a lot of effort to be healthy”*.

#### *Environmental level*

When asking participants for suggestions regarding intervention development, students believed that the student restaurant could provide more healthy menus: *“The student restaurant should offer more healthy menu choices, so you actually oblige students subtly to eat healthy” (…) “It would be good when, for example, they (the student restaurant) wouldn’t always offer French fries, because (when available) I tend to choose French fries very often”*. Concerning price and cost, it was also mentioned that *“the student restaurant should modify its prices, because that would motivate students to eat more healthy foods when lower in price” (…) “Students will choose a healthy menu (e.g. vegetarian pasta) lower in price over a less healthy and more expensive one (e.g. steak). At least, I know I would, although normally, I would rather eat meat”*. Participants also expressed that *“they (the government) should implement higher taxes for unhealthy (e.g. high-fat) foods”*. Another suggestion was to display the amount of calories on every menu. *“When the student restaurant would display calories, lots of students will probably think twice when choosing a dish.”* Students also felt that campus vending machines should contain more healthy products: *“The on-campus vending machines are not healthy” (…) “In secondary school, they replaced all vending machine products by healthy foods and it led to lower consumption of vending products, but also, students were obliged to choose a healthy product”*.

## Discussion

The purpose of this explorative study was to identify determinants of eating (incl. drinking) behaviours in Belgian (European) university students. Furthermore, we aimed to collect ideas and recommendations in order to facilitate the development of effective and tailored intervention programs aiming to improve healthy eating (incl. drinking) behaviours in university students. Similar to Story’s framework [19] combining Bandura’s Social Cognitive Theory [15] with Sallis’ ecological model [21] explaining health behaviour, we identified four major levels of determinants: individual (intrapersonal), social environment (interpersonal), physical environment (community settings) and macro environment (societal). Furthermore, some university specific characteristics were found to be influencing students’ eating behaviours as well.

Similar to US literature [22-25], many self-regulatory processes, including intrinsic (e.g. food preferences) and extrinsic (e.g. health awareness, guilt) motivations, self-discipline, self-control, time management, etc. have been mentioned by our participants to be influencing eating behaviour in university students. Our results further indicate that these latter determinants become more important after the transition from secondary school to university when independency subsequently increases. In a qualitative study of Cluskey et al. [25], university students who reported greater independency and more responsibility for food and meal preparation prior to college, felt to have achieved more stability in their eating behaviours at college. Therefore, LaCaille et al. [23] suggested that future interventions should aim at strengthening students’ self-regulation skills around eating as part of the overall transition to university or college. Such self-regulation and self-management skills can help students to make more healthy decisions and to maintain a healthful lifestyle throughout adulthood [24]. Moreover, the systematic review of Kelly et al. [29] evaluating the effectiveness of dietary interventions in college students suggested that approaches involving self-regulation strategies have the potential to facilitate changes in students’ dietary intake.

Although in the study of Cluskey et al. [25] US students most agreed that intrinsic motivation was needed for successful changes in healthful behaviour, our results indicate that the environment should be organised as such, ‘forcing’ students to make healthful food choices. Students’ food choices are influenced by the availability and accessibility of healthy foods and cooking supplies [22]. Therefore, our participants suggested that offering more healthy menus in the student restaurant as well as providing campus vending machines with more healthy products could contribute to making more healthful food choices. It has been shown that food availability and accessibility of fruits and vegetables is strongly and positively related to fruit and vegetable consumption in children [30]. In addition, students in the current study mentioned that the appeal of (on-campus) foods often determines food choices. This suggests that making healthy products offered around campus more appealing might contribute to more healthy eating behaviours in university students.

Students in the current study believed they are continuously challenged by competing demands, including academic responsibilities and involvement in extracurricular and social activities. As mentioned by Nelson et al. [24], healthy food choices may become low priorities when compared to other commitments. Therefore, as described by our participants, students might be more likely to buy foods that are fast, convenient and inexpensive. Marquis et al. [31] showed that college students often prioritize cost and convenience over health. Moreover, previous studies found that price is one of the most influential individual factors (next to taste) in determining food choice in both adults and adolescents [32-36]. In our study, participants felt that offering more healthy (on campus) foods at a lower cost would contribute to more healthful food choices. Intervention studies in other populations have shown that price reductions increase purchases of lower-fat products and fruits and vegetables in cafeterias, workplaces and school vending machines [34,36]. Given the importance of price in university students’ food choices, this might even be a more effective strategy in this population.

Similar to previous research in adolescents [37], participants felt that perceived benefits (e.g. improving health status, higher vitality) of healthful eating behaviour can influence food choices as well. Students also believed that dietary knowledge should be a first step towards the awareness on healthy eating behaviour. Cluskey et al. [25] mentioned that university students lack the knowledge and skills to make healthful food choices as well as to prepare healthy foods, which makes it difficult to adapt healthfully to college or university life. It was suggested by our participants that all students should be given a health education class.

In this study, students mentioned that parents and household influence their food intake. A review study on environmental influences on food choices [38] indicated that adolescents’ dietary intake is being influenced by their family members. Parents serve as models for eating behaviour and transmit dietary attitudes throughout the upbringing of their offspring [38]. The latter suggests that especially university students living with their parents might experience similar parental influences. Besides family influences, our participants believed that friends and peers influence their eating behaviour as well. Contento et al. [39] reported that attitudes, encouragement, and behaviours of friends and peers influenced adolescents’ food choices. In a natural experiment assessing peer effects on weight, it was shown that the amount of weight gained during the freshman year was strongly and negatively correlated to the roommate’s initial weight [40], suggesting that peers are influenced by each other’s eating behaviours.

Living arrangements (residency) and exams were mentioned to be influencing students’ eating behaviours. It has been shown that living arrangements can affect university students’ dietary intake [41-43]. In a study in four European countries students living at parental home consumed more fruit and vegetables than those who resided outside of their family home [43]. In addition, in a natural experiment, Kapinos et al. [44] showed that students assigned to dormitories with on-site dining halls gained more weight and exhibited more behaviours consistent with weight gain (e.g. males consumed more meals and snacks) during the freshman year as compared with students not assigned to such dormitories. Living arrangements might be moderating the relation between eating behaviour and its determinants rather than causing eating behaviour as such. According to MacKinnon [45], a moderator affects the strength of the relation between two variables. E.g. living in a student residence may moderate the relation between parental influence and eating behaviour, i.e. parental control will decrease when students live away from home. Results of the current study suggest that the relation between parental control and eating behaviour might be stronger in students living at home compared to those living away from home. Also, when living in a student residence (and receiving a weekly based allowance) our results revealed that food prices become more important when making food choices, i.e. students have to pay attention to ‘what’ they buy. Thus, a stronger relationship between food prices and eating behaviour might be observed when students live away from home in comparison to those living with their parents. Exams can have a similar moderating effect on the relation between e.g. time and eating behaviour. Our results show that during exam periods students will spend as little time as possible on cooking.

When comparing with the limited US literature, it should be noticed that, despite similarities between this study and other US studies (e.g. lack of time, unorganised living), some determinants can be region or culture-specific. For example, students in the present study referred to the abundant availability of fast food and one-dollar-menus in the US, in comparison to Europe. Focus group discussions with US university students pointed out that all-you-can-eat formulas of on-campus dining facilities had a negative impact on their healthy eating behaviours [23,25]. In contrast to US universities our universities do not dispose of all-you-can-eat dining possibilities. Furthermore, unlike US colleges/universities, Belgian universities do not have on-campus dormitory dining halls where campus meal plans for students are provided.

Our results also indicate that what may be a barrier for one student may be perceived as an enabler by another. For example, with regard to the physical campus environment, some students felt that the student restaurant was a barrier to healthful eating behaviour, whereas others believed it enabled students to make healthy food choices. Therefore, with regard to future intervention programs, we should modify perceptions of physical environment as well, rather than the objective environment on its own.

Furthermore, our results indicate that physical and social environments are continuously interacting with self-regulatory processes and thus individual eating behaviours. It could be that a certain stimulation at the individual level might not be changing one’s eating behaviour when acting in a non-beneficial social and/or physical environment, and vice versa. Therefore, intervention strategies based on multilevel approaches may be most effective [21].

This qualitative research methodology, using focus groups, is an important strength of this explorative study. As Sallis et al. [46] suggested, qualitative research allows us to understand not only the ‘what’ but also the ‘how’ and ‘why’. Using an inductive thematic methodology allowed the research team to construct a student-specific framework. Furthermore, in contrast to in-depth interviews, the more ‘naturalistic’ approach (i.e. closer to everyday conversation), including dynamic group interaction [28], allowed us to get better insight into the mechanisms behind university students’ eating behaviours. On the other hand, some participants might have been intimidated by the group setting which might have limited a greater sharing of their thoughts.

A first limitation of this study is that we used student volunteers. We have to take into account that participants were probably interested in this topic, which might have resulted in a selection bias. However, sample characteristics showed sufficient variety in BMI and perceived health status. Secondly, whereas we might expect differences in behaviours according to gender [47] or year in school, we chose to use mixed-gender focus groups including students of different study years and disciplines, allowing us to create interaction between both genders with a variety of study experience and backgrounds, which in turn generated a greater diversity in opinion within each focus group. Thirdly, focus groups were conducted at one university, which has a campus outside the city centre. Because of university specific environmental differences (e.g. size, structure, region, etc.), the applicability of the study’s findings to other student populations is limited to the psychosocial level, whereas future studies should further explore the physical environmental issues within a variety of other Belgian or European universities. Finally, because of the abovementioned setting limitation and the explorative nature of this study no conclusions can be drawn concerning the importance of each determinant and the generalizability of our results. The purpose of using focus groups is to generate a rich understanding of participants’ experiences and beliefs [48,49] and not to generalize results [50]. In addition, no quantification was used because numbers and percentages convey the impression that results can be projected to a population, and this is not within the capabilities of qualitative research [50]. Also, the issue raised most frequently is not necessarily the most important, even when it is raised by a larger number of people [50]. In other words, each idea or opinion should be equally appreciated. Therefore, future studies, using a larger representative sample size, should focus on providing quantitative evidence regarding the importance and value of each determinant, making it also possible to differentiate according to gender, year in school, study discipline, or other student characteristics. Subsequently, future tailored interventions could focus on those factors students experience as most determinative in their current eating behaviour.

## Conclusions

To the best of our knowledge, this is the first European study examining perceived determinants of eating (incl. drinking) behaviour in university students and collecting ideas and recommendations in order to facilitate the development of effective and tailored intervention programs aiming to improve healthy eating (and drinking) behaviours in university students. An ecological framework of determinants of university students’ eating behaviour was developed. Students were found to be influenced by individual factors, their social networks, physical environment, and macro environment. Furthermore, the relationships between determinants and university students’ eating behaviour seemed to be moderated by university characteristics, such as residency, student societies, university lifestyle and exams. After the transition from secondary school to university, when independency increases, students are continuously challenged to make healthful food choices. They have to be self-disciplined, have self-control and thus often have to prioritize healthy eating over other (university specific) social activities in order to prepare a healthy meal. In addition, students have to make these healthful food choices within a university specific setting (e.g. living in a student residence, having exams), depending on the availability and accessibility, appeal and prices of food products. Moreover, during this choice making process, students are either controlled or lacking control by their parents as well as influenced by friends and peers. Recommendations for university administrators and researchers include providing information and advice to enhance healthy food choices and preparation (e.g. via social media), enhancing self-discipline and self-control, developing time management skills, enhancing social support, and modifying the subjective as well as the objective campus food environment by e.g. making healthy foods price-beneficial and by providing vending machines with more healthy products. Our results should be considered a first step into the development of tailored and effective intervention programs aiming to improve university students’ eating behaviours.

## Abbreviations

BMI: Body mass index; SD: Standard deviation; yrs: Years.

## Competing interests

The authors declare that they have no competing interests.

## Authors’ contributions

TD participated in the design of the study, collected all data, performed the data analyses and drafted the manuscript. IDB participated in the design of the study and revised the manuscript critically. PC and BD participated in the design of the study, contributed to the interpretation of data and revised the manuscript critically. All authors read and approved the final manuscript.

## Pre-publication history

The pre-publication history for this paper can be accessed here:

http://www.biomedcentral.com/1471-2458/14/53/prepub
